# Weekly intra-articular injections of bone morphogenetic protein-7 inhibits osteoarthritis progression

**DOI:** 10.1186/ar2521

**Published:** 2008-09-30

**Authors:** Masaya Hayashi, Takeshi Muneta, Young-Jin Ju, Tomoyuki Mochizuki, Ichiro Sekiya

**Affiliations:** 1 Section of Orthopaedic Surgery, Graduate School, Tokyo Medical and Dental University, Yushima, Bunkyo-ku, Tokyo, 113-8519 Japan; 2 Section of Cartilage Regeneration, Graduate School, Tokyo Medical and Dental University, Yushima, Bunkyo-ku, Tokyo, 113-8519 Japan

## Abstract

**Introduction:**

We investigated the ability of a weekly intra-articular injection of bone morphogenetic protein (BMP)-7 to prevent osteoarthritis in rabbits with anterior cruciate ligament transections.

**Methods:**

First, 36 knee joints were randomly divided into four groups: 50, 500, 5,000 ng BMP-7, and control. Knee cartilage was evaluated at 4, 8, and 12 weeks. Then, in order to control for individual differences, 500 ng BMP-7 was injected into one knee and phosphate-buffered saline (PBS) into the other, and the two knees were compared at 4, 8, and 12 weeks (*n* = 5). For pharmacokinetic analysis, cartilage was harvested at 1 hour and 1, 2, 4, and 7 days after knee injection of 500 ng BMP-7 or PBS (*n* = 3).

**Results:**

Histological scores in the 500 and 5,000 ng BMP-7 groups were significantly better than those in the other groups at 12 weeks. Matched pair analysis demonstrated that both macroscopic and histological scores in the 500 ng BMP-7 group were better than those in the control group. Immunohistochemical analysis revealed higher BMP-7 expression by chondrocytes in the BMP-7 injected knees. Histology of whole knee and quantitative micro computed tomography analysis showed that weekly injections of 500 ng BMP-7 did not induce synovial fibrosis, ectopic bone, or osteophyte formation. As detected by enzyme-linked immunosorbent assay, BMP-7 concentration in the cartilage tissue was still higher in the BMP-7 treated group 7 days after the injection.

**Conclusions:**

Weekly intra-articular injections of BMP-7 inhibited progression of osteoarthritis. Obvious adverse effects were not observed. BMP-7 concentration and expression in cartilage were still higher 7 days after injection.

## Introduction

Osteoarthritis of the knee is one of the leading causes of disability among elderly people. It is mainly caused by the breakdown and eventual loss of joint cartilage. For many years, scientists have been searching for ways to intervene in the disease process and so retard or even prevent progression of joint damage.

Bone morphogenetic protein (BMP)-7, also referred to as osteogenic protein-1, has a profound effect on chondrocyte metabolism by stimulating the synthesis [1-3], organization [4], and retention [2,5] of matrix molecules. With increasing age and progression of articular cartilage degeneration, the expression level of endogenous BMP-7 decreases [6], suggesting that a decrease in BMP-7 may play an important role in the progression of cartilage degeneration. A recent study [7] demonstrated that continuous intra-articular infusion of BMP-7 had a protective effect on cartilage degeneration, which suggests the possible utility of BMP-7 as a treatment for human osteoarthritis.

We speculated that periodic knee injections of a small amount of BMP-7 would suppress the loss of cartilage matrix and consequently prevent osteoarthritis progression, without any adverse drug effects. In this study, BMP-7 was injected weekly into the knee joints of rabbits after anterior cruciate ligament transection (ACLT), and the cartilage of the knee was evaluated morphologically. The knees were also evaluated for any possible adverse effects of the BMP-7. Furthermore, BMP-7 concentration in cartilage was sequentially analyzed after intra-articular injection of BMP-7. The overall results suggest that weekly intra-articular injection of a small amount of BMP-7 is a promising nonsurgical treatment for osteoarthritis, and that BMP-7 is an interesting candidate structure/disease-modifying osteoarthritis drug.

## Materials and methods

### Animals and injection of BMP-7

Skeletally mature female Japanese white rabbits (10 ± 2 months old) weighing an average of 3.2 kg (range 2.8 to 3.6 kg) were used in the experiments. This study was conducted in accordance with a protocol approved by the Animal Committee of Tokyo Medical and Dental University. Animals underwent bilateral ACLT under anesthesia induced by intramuscular injection of 25 mg/kg ketamine hydrochloride (Sankyo, Tokyo, Japan) and intravenous injection of 45 mg/kg sodium pentobarbital (Dainippon Sumitomo Pharma, Osaka, Japan). The knee joint was approached through a medial parapatellar incision, and the patella was dislocated laterally. The anterior cruciate ligament was then transected with a sharp blade, and the capsule was sutured to render it watertight, followed by skin closure. All animals were allowed normal cage activity.

Lyophilized 5% lactose-buffered recombinant human BMP-7 (rhBMP-7; Stryker Biotech, Hopkinton, MA, USA) was dissolved in phosphate-buffered saline (PBS). Aliquoted 50, 500, or 5,000 ng BMP-7 in 200 μl PBS was administrated intra-articularly with a 27-gauge needle on a 1.0 ml syringe through the lateral infrapatellar area toward the intercondylar space of the femur in each animal in a deep knee-flexed position. The first injection was given immediately after ACLT; the second and subsequent injections were administered once a week up to 12 weeks. The final injection was administered a week before the animals were killed, by an overdose of sodium pentobarbital; the knee joints were then evaluated.

For evaluation of the optimal dose of BMP-7, 18 rabbits with 36 knees were used. All animals underwent bilateral ACLT. The 36 knee joints were randomly assigned to one of four groups: three doses (50, 500, or 5,000 ng) of BMP-7 or control (PBS alone).

For matched pair analyses of BMP-7, 15 rabbits with 30 knees were used. The other three rabbits with six knees were used for whole knee experiments conducted to investigate the intra-articular influences of BMP-7. The dose of 500 ng was chosen based on the previous results. In each of the 18 animals, BMP-7 was injected into the right knee and PBS into the left, as a control.

### Gross morphological examination

Femoral condyles were dissected and stained with India ink. Macroscopic pictures were taken using MPS-7 (Sugiura Laboratory Inc., Tokyo, Japan), a dedicated medical photography platform, and used for macroscopic evaluation. Digital images were taken using a Nikon Coolpix 4500 digital camera (Nikon, Tokyo, Japan). Gross findings were classified into six grades (grade 1: intact articular surface; grade 2: minimal fibrillation; grade 3: overt fibrillation; grade 4a: erosion of 0 to 2 mm; grade 4b: erosion of 2 to 5 mm; and grade 4c: erosion of >5 mm) and scored accordingly [8]. Both the medial and lateral femoral condyles were individually scored. Then, the two scores were summed to obtain a cumulative macroscopic osteoarthritis score. In a blinded manner, the assessment was conducted by two independent examiners, who were blinded to each other's findings and to the treatment group assignment of the animals. Finally, the two scores from the examiners were averaged to obtain an overall score.

### Histological examination

The dissected distal femurs were fixed in a 4% paraformaldehyde solution after gross morphological examination. The specimens were decalcified in 4% EDTA solution, dehydrated with a gradient ethanol series, and embedded in paraffin blocks. Based on macroscopic observation, 20 coronal sections per knee were carefully prepared so as to include the most severely degenerated area. For whole knee specimens, sagittal sections were stained with Masson's trichrome. Histological sections were visualized using an Olympus IX71 microscope (Olympus, Tokyo, Japan) and PIXERA Viewfinder 3.0 software (Pixera Corporation, San Jose, CA, USA). Histological sections were assessed in a blinded manner by two individual examiners, who were unaware of the treatment group assignment of the animals, and quantified using the advanced grading methodology of the Osteoarthritis Research Society International (OARSI) osteoarthritic cartilage histopathology grading system [9].

### Immunohistochemical analysis

Paraffin-embedded sections were deparaffinized in xylene, rehydrated through graded alcohol, and immersed in PBS. The samples were pretreated with 0.4 mg/ml proteinase K (DAKO, Carpinteria, CA, USA) in Tris-HCl buffer for 15 minutes at room temperature for antigen retrieval. Any residual enzymatic activity was removed by washing with PBS, and nonspecific staining was blocked by preincubation with PBS containing 10% normal horse serum for 20 minutes at room temperature. Mouse monoclonal anti-BMP-7 antibody (12G3; 1:100 dilution; Stryker Biotech, Hopkinton, MA, USA) was placed on the sections overnight at 4°C. After extensive washing with PBS, the sections were incubated in the secondary antibody of biotinylated horse anti-mouse IgG (Vector Laboratories, Burlingame, CA, USA) for 30 minutes at room temperature. Immunostaining was detected with VECTASTAIN ABC reagent (Vector Laboratories), followed by DAB staining. Counterstaining was performed with methyl green.

### Semi-quantitative analysis of synovial fibrosis

The whole knee specimens stained with Masson's trichrome were analyzed in order to measure the rate of synovial fibrosis in the infrapatellar fat pad (IPF) [10,11]. The area of the whole IPF and blue stained area of collagen fibers was measured using Scion Image software (Scion Corporation, Frederick, MD, USA). The rate of fibrosis in IPF (% fibrosis) was calculated as the blue stained area divided by the whole IPF area × 100.

### Micro computed tomography scanning and quantification of osteophyte volume

All specimens were subjected to analysis using a high-resolution micro computed tomography scanner (ScanXmate-E090; Comscantecno, Kanagawa, Japan). Osteophytes were manually traced and detected in 10 axial sections, interpolated, reconstituted, and quantified using TRI/3D-BON software (RATOC, Tokyo, Japan).

### Pharmacokinetic analysis of BMP-7 in cartilage tissue

At 1 hour and 1, 2, 4, and 7 days after intra-articular injection of 500 ng BMP-7 or PBS into normal knees, rabbits were killed, and cartilage tissue from their knee joints was collected. The cartilage was homogenized in CelLytic™ MT Mammalian Tissue Lysis/Extraction Reagent (Sigma, St. Louis, MO, USA) and the lysed samples were centrifuged for 10 minutes at 12,000 *g*. The supernatant was stored with protease inhibitor cocktail (Sigma, St. Louis, MO, USA) and BMP-7 levels were measured using a sandwich enzyme-linked immunosorbent assay. Monoclonal 1B12 antibody (Stryker Biotech, Hopkinton, MA, USA) was utilized as a coating antibody. Plates were coated with 1 μg/well of this antibody in sodium carbonate-bicarbonate buffer and incubated overnight at 4°C. Nonspecific binding was blocked with 4% milk/borate-buffered saline containing 0.05% Tween-20 (BBST) blocking buffer. Either BMP-7 standard or cartilage extract was added to the plate and incubated at 37°C for 1 hour. A second anti-BMP-7 antibody labeled with alkaline phosphatase (12G3-AP) was applied at 1:100 dilution in sodium carbonate-bicarbonate buffer and incubated at 37°C for 1 hour. After this incubation, an alkaline phosphatase substrate, PNPP-phosphatase substrate (Pierce, Rockford, IL, USA), was added and incubated at room temperature for 30 minutes. To stop the reaction, 2 N NaOH was added. The absorbance was detected and quantitated by microplate reader Sunrise Remote (TECAN, Männedorf, Switzerland). The data were then processed in LS-PLATEmanager software (Wako, Osaka, Japan).

### Statistical analysis

All data are expressed as mean ± standard deviation. A nonparametric Mann-Whitney U test was used to evaluate the statistical significance of differences in the macroscopic and histologic results. A Wilcoxon's signed rank-sum test was used to perform matched pair analyses. Interobserver variation in gross morphologic grading was verified by measuring agreement with the κ statistic. *P *values less than 0.05 were considered statistically significant.

## Results

### Dose effect of BMP-7 for preventing the progression of osteoarthritis

**Figure 1:**
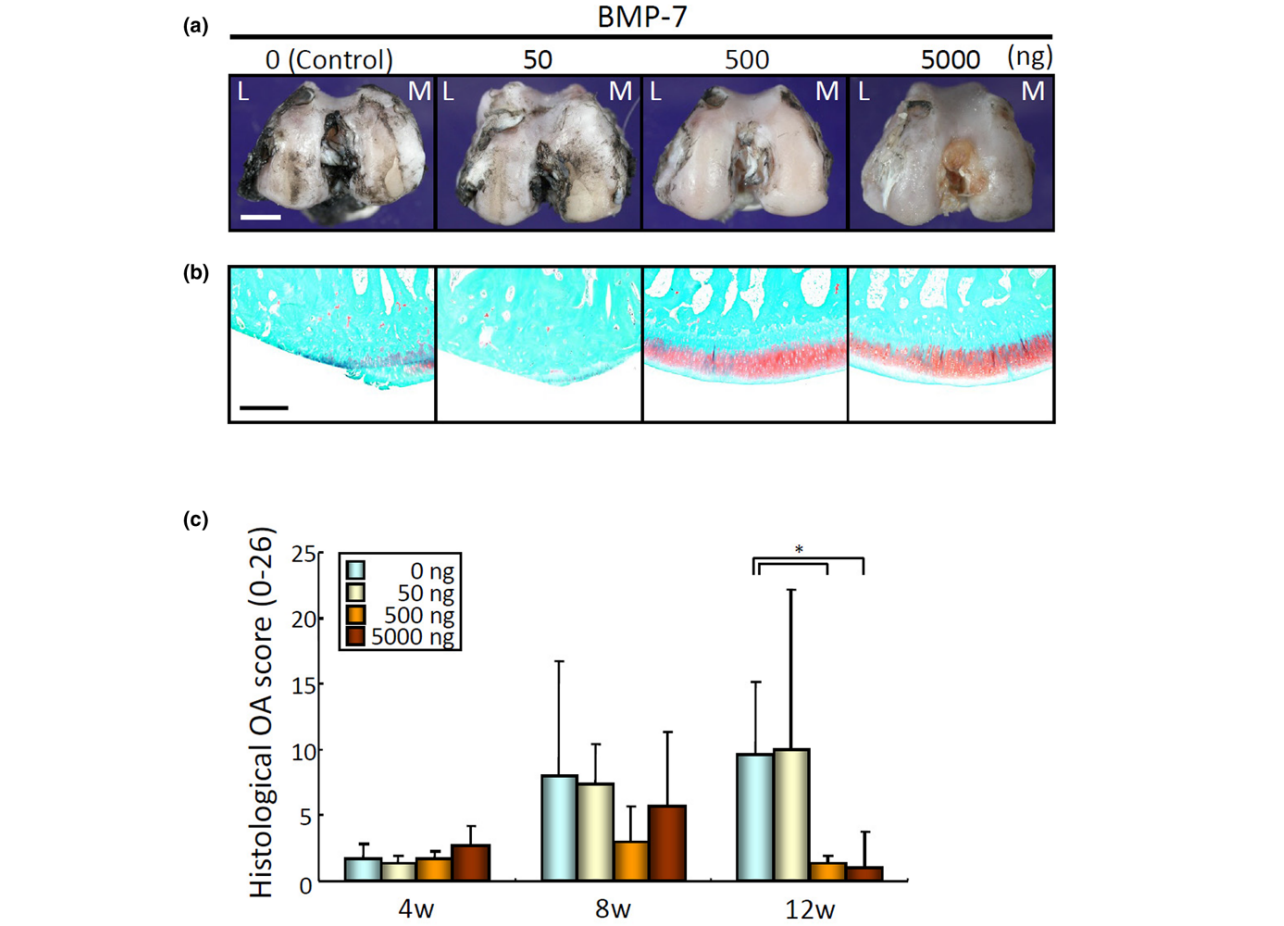
Evaluation of dose effect of BMP-7 for prevention of osteoarthritis progression in rabbit ACLT. **(a) **Representative macroscopic appearances of the distal portion of the femoral condyles at 12 weeks. Surface of the cartilage was stained with India ink to identify any fibrillation and erosion. Laterality is shown as lateral (L) and medial (M). Bar = 5 mm. **(b) **Representative histology of femoral condyles at 12 weeks. Distal femur was sectioned coronally and stained with safranin-O. The most degenerated area of each sample is included. Bar = 50 μm. **(c) **Quantitation of histological analysis using the OARSI cartilage osteoarthritis histopathology grading system. The scores are displayed as average ± standard deviation (three knees). *P < 0.05, by Mann-Whitney U test. ACTL, anterior cruciate ligament transection; BMP, bone morphogenetic protein; OARSI, Osteoarthritis Research Society International.

After ACLT, 50, 500, or 5,000 ng BMP-7 in 200 μl PBS or PBS alone was injected weekly into the knee joint. Macroscopic observation of femoral condyles at 12 weeks demonstrated obvious surface irregularity in the control and 50 ng BMP-7 groups, whereas a milder alteration in the articular surface was observed in the 500 ng and 5,000 ng BMP-7 groups (Figure 1a). No damage was caused by the needle. Histologically, cartilage matrix disappeared in the control and 50 ng BMP-7 groups, whereas it was predominantly retained in the 500 ng and 5,000 ng BMP-7 groups (Figure 1b). The OARSI osteoarthritis scores for histologic analysis were similar in all four groups at 4 and 8 weeks; however, they were significantly better in the 500 ng and 5,000 ng BMP-7 groups than in the control group at 12 weeks (Figure 1c). These data indicate that weekly injections of 500 ng and 5,000 ng BMP-7 prevented the progression of osteoarthritis. There were no significant differences between the 500 ng and 5,000 ng groups. The dose-response effect of BMP-7 reached a plateau at 500 ng, and therefore the dose of 500 ng BMP-7 was used in further analyses.

### Matched pair analyses: effect of BMP-7 on osteoarthritis progression

During the early stage of the investigation, we found that rabbits exhibited considerable individual variability in osteoarthritis progression after ACLT. To examine the effect of BMP-7 on osteoarthritis progression in a stricter manner, after the ligaments in the both knees were dissected, 500 ng BMP-7 in 200 μl PBS was injected into the right knee, and the same amount of PBS was injected into the left knee of the same animal on a weekly basis.

**Figure 2:**
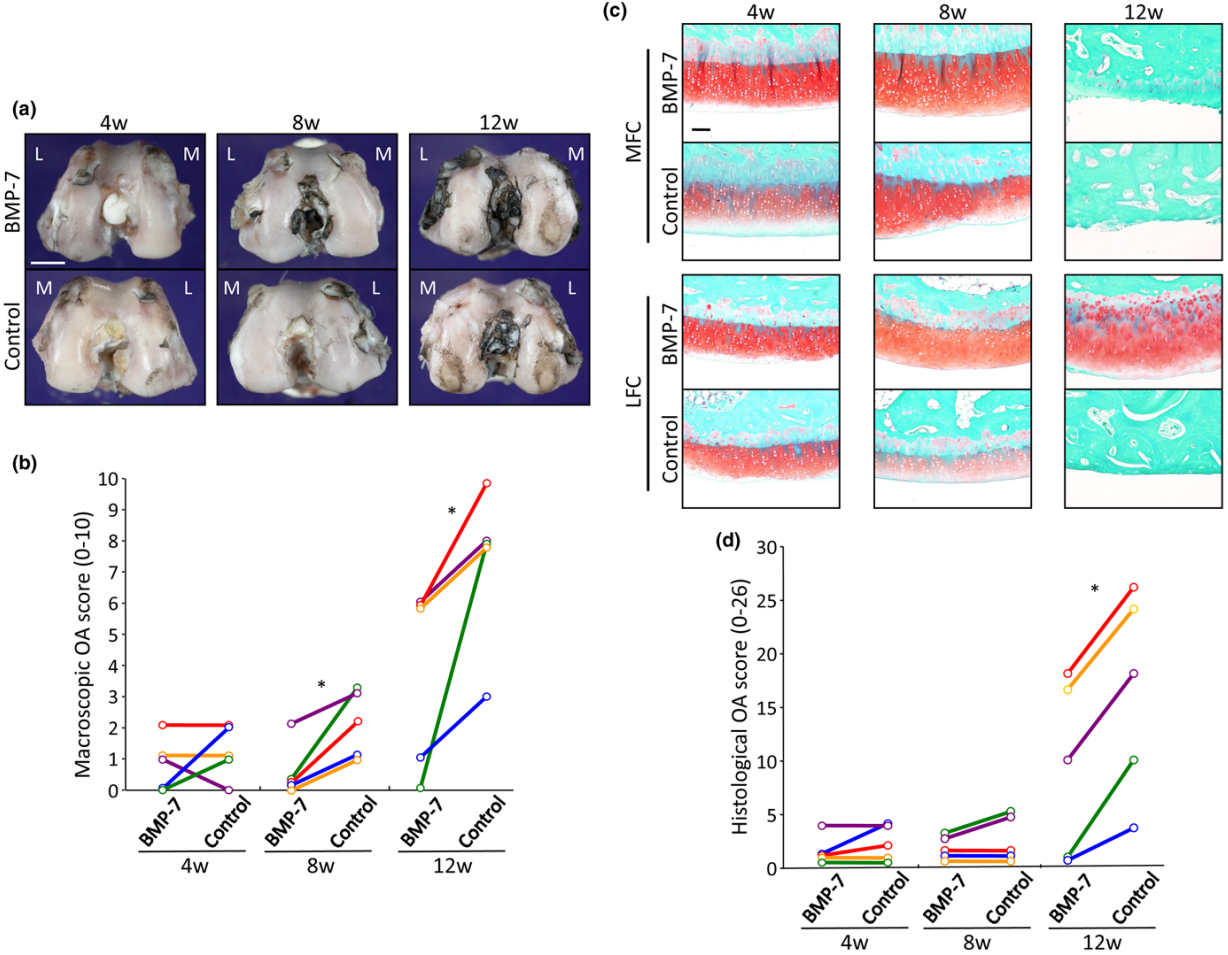
Matched pair analyses of the effect of BMP-7 on osteoarthritis progression. **(a) **Macroscopic appearances of the femoral condyles at 4, 8, and 12 weeks. To remove individual variability, both sides of the knees of the same individuals are shown. Surface of the cartilage was stained with India ink to identify any fibrillation and erosion. Laterality is shown as lateral (L) and medial (M). Bar = 5 mm. **(b) **Paired comparison in the macroscopic osteoarthritis score. The scores of the treatment and control knees (in the same animal) are displayed separately and connected with a line (five knees). *P < 0.05, by Wilcoxon's signed rank sum test. **(c) **Representative histology of femoral condyles. Both sides of the knees from the same individuals are shown. Medial femoral condyles (MFC) and lateral femoral condyles (LFC) were sectioned coronally and stained with safranin-O. Bar = 10 μm. **(d) **Paired comparison in quantitation of histological analysis using the OARSI cartilage osteoarthritis histopathology grading system (five knees). *P < 0.05 by Wilcoxon's signed rank sum test for paired samples. BMP, bone morphogenetic protein; OARSI, Osteoarthritis Research Society International.

Macroscopic observations on gross morphologic changes of the femoral condyles in the control group revealed subtle cartilage lesions at 4 weeks, a slight progression at 8 weeks, and obvious surface irregularity at 12 weeks (Figure 2a). On the contrary, cartilage of the femoral condyles in the treatment group appeared to be better throughout the study. Interestingly, four out of five rabbits in the control side/group exhibited erosions in both the lateral and medial femoral condyles at 12 weeks; however, in the 500 ng BMP-7 side/group, only two rabbits exhibited erosions in the lateral femoral condyle, and two other rabbits showed erosions in the medial femoral condyle. In all animals, the macroscopic osteoarthritis score was better in the BMP-7 injected knee than in the contralateral control knee (Figure 2b). The interobserver agreement for grading of cartilage damage indicates high reproducibility (κ = 0.84).

Histologically, stainability with safranin-O in cartilage matrices appeared to be similar in both the 500 ng BMP-7 and control groups at 4 weeks (Figure 2c). At 8 weeks, in the control side/group, cartilage became thinner or stainability with safranin-O became worse than in the BMP-7 side/group. At 12 weeks, cartilage lesions further worsened, and most of the knees in the control side/group exhibited severe erosion or cartilage defects in both the medial and lateral condyles. BMP-7 injected knees also developed erosive lesions; however, those were limited either to the medial or to the lateral femoral condyle. The OARSI osteoarthritis score was better at the BMP-7 side than at the contralateral control side in each rabbit at 12 weeks (Figure 2d). Matched pair analyses revealed that weekly 500 ng BMP-7 injections slowed the progression of osteoarthritis.

### BMP-7 expression in cartilage of knee after weekly BMP-7 injection

**Figure 3:**
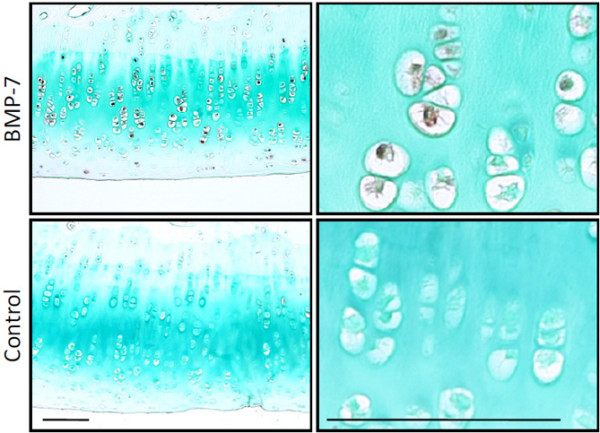
Immunohistochemical analysis for BMP-7 in cartilage. After anterior cruciate ligament transection in both knees, 500 ng BMP-7 in 200 μl PBS was injected weekly into the right knee and the same amount of PBS was injected into the left knee. The remaining cartilage in medial femoral condyles was evaluated at 12 weeks (7 days after the last injection). Higher magnifications are demonstrated in the right column. Bars = 10 μm. BMP, bone morphogenetic protein; PBS, phosphate-buffered saline.

Immunohistochemical analysis showed that BMP-7 was barely expressed in chondrocytes of the remaining cartilage in the control knees. On the contrary, the rate of chondrocytes staining positive for BMP-7 was higher in the knees receiving treatment with BMP-7 despite 7 days having passed since the last injection (Figure 3).

### Investigation of presumable adverse effects of BMP-7

It was hypothesized that repeated BMP-7 injections into the knee joint might induce adverse effects such as synovial fibrosis, ectopic cartilage and bone formation, or osteophyte formation. Various techniques were used to screen for any possible unwanted response.

**Figure 4:**
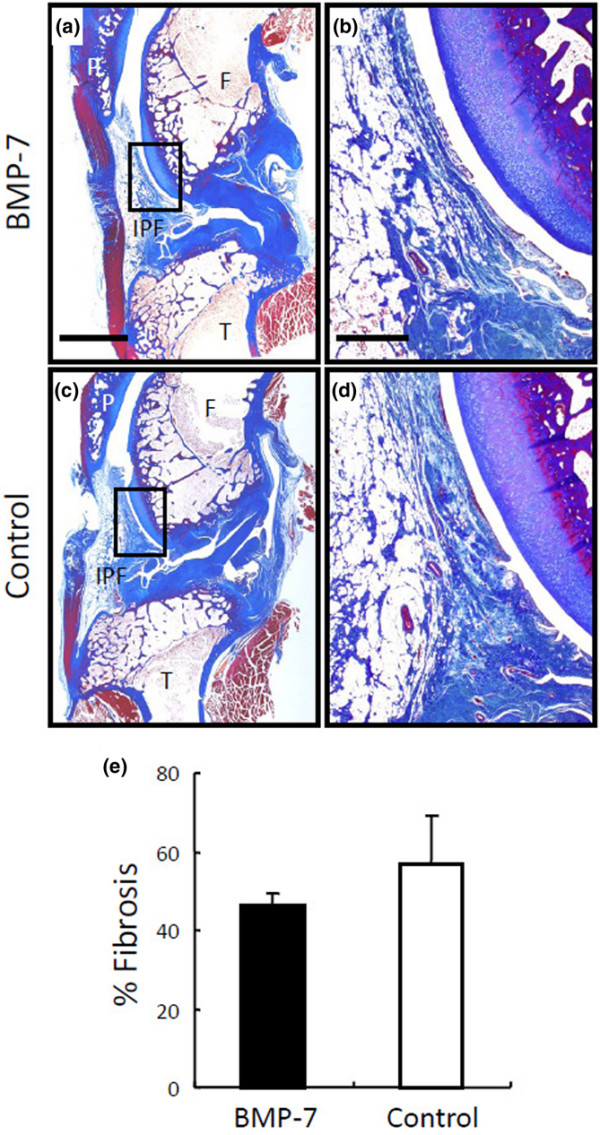
Evaluation of synovial fibrosis. After anterior cruciate ligament transection in both knees, 500 ng BMP-7 in 200 μl PBS was injected weekly into the right knee and the same amount of PBS was injected into the left knee. Whole knee sections stained with Masson's trichrome were evaluated at 12 weeks after ACLT. **(a, c) **Lower magnified histologies. Bar = 5 mm. **(b, d) **Higher magnified histologies of infrapatella fat pad. Bar = 1 mm. **(e) **Quantitation of fibrosis in infrapatellar fat pad. The values are displayed as average ± standard deviation (*n* = 3). BMP, bone morphogenetic protein; F, femur; IPF, infrapatellar fat pad; P, patella; PBS, phosphate-buffered saline; T, tibia.

**Figure 5:**
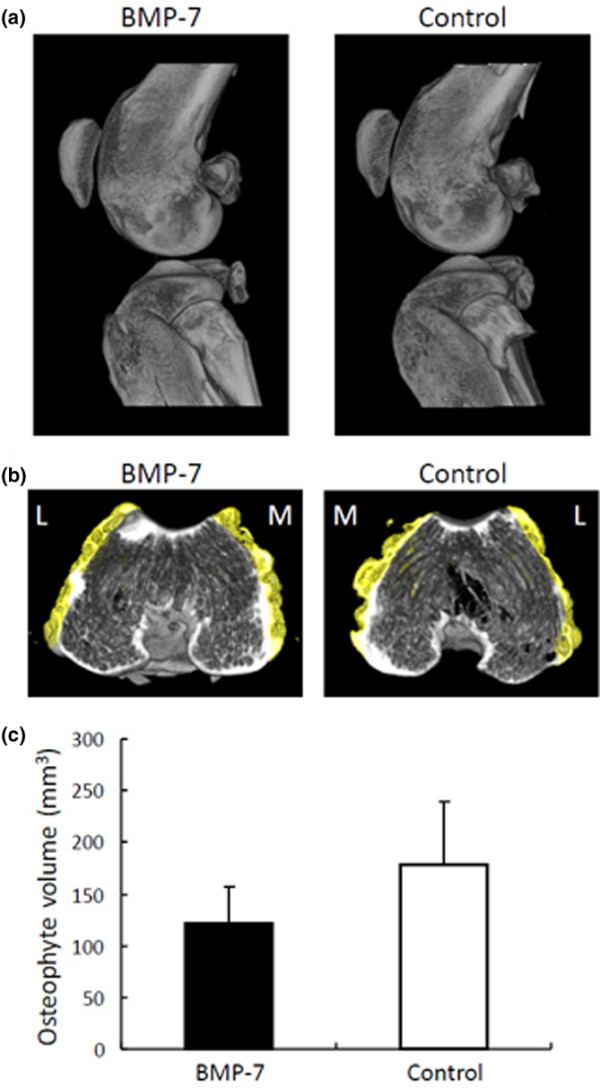
Evaluation of ectopic bone and osteophyte formation. After anterior cruciate ligament transection in both knees, 500 ng BMP-7 in 200 μl PBS was injected weekly into the right knee and the same amount of PBS was injected into the left knee. Knee joints were analyzed using a micro CT scanner at 12 weeks. **(a) **Representative three-dimensional CT images of BMP-7 treated and control knee. Lateral views show no obvious ectopic bone formation in BMP-7 injected side and control side. **(b) **Representative reconstructed CT images of distal femoral osteophyte. Osteophytes were colored with yellow by image processing. Laterality is shown as lateral (L) and medial (M). **(c) **Quantitation of osteophyte volume. The values are displayed as average ± standard deviation (*n* = 5). BMP, bone morphogenetic protein; CT, computed tomography; PBS, phosphate-buffered saline.

For synovial fibrosis, fibrotic areas in the IPF were compared. According to the quantitative analysis, the ratio of fibrotic area in the BMP-7 treated knee was not higher than that in the control knee (Figure 4). We examined the area around the border between cartilage and synovium, the area around the sutured capsule, and the area around the dissected anterior cruciate ligament carefully, but we did not detect ectopic cartilage formation in the knees injected with BMP-7. For ectopic bone or osteophyte formation, whole knee joints were evaluated by micro computed tomography. No ectopic bone formation was observed by three-dimensional reconstructed imaging (Figure 5a). Quantitative analysis showed that the volume of osteophyte formation in the BMP-7 group was not more than that in the control group at 12 weeks (Figure 5b,c). Based on these results, it appears that BMP-7 did not induce any obvious adverse effects.

### Pharmacokinetic analysis of BMP-7 in cartilage tissue

**Figure 6:**
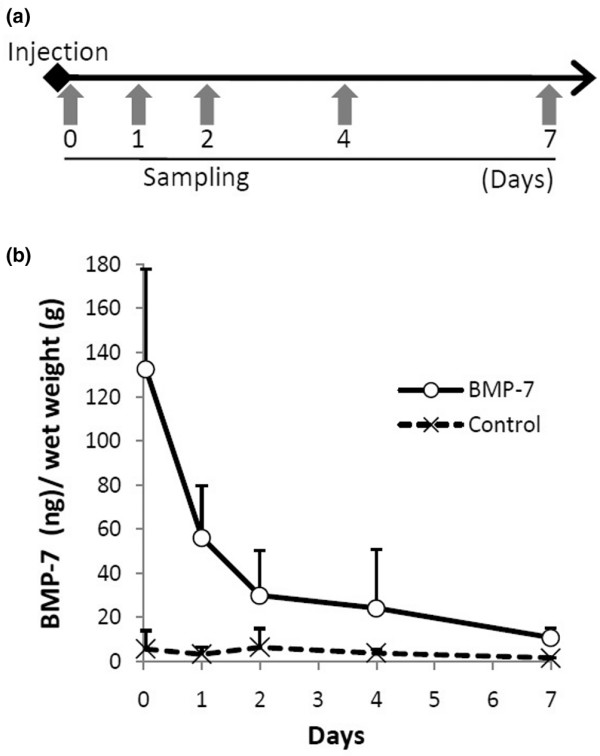
Pharmacokinetic analysis of BMP-7 in cartilage tissue. **(a) **Outline for the analysis. Cartilage was obtained from knee joints at 1 hour and 1, 2, 4, and 7 days after intra-articular injection of 500 ng BMP-7 or phosphate-buffered saline. Three joints for each time point were investigated. **(b) **BMP-7 concentration per cartilage wet weight (ng/g). The values are shown as average ± standard deviation. BMP, bone morphogenetic protein.

BMP-7 levels in cartilage tissue were measured using sandwich enzyme-linked immunosorbent assay (Figure 6a). In control knees, BMP-7 was barely detected at any of the observation times (Figure 6b). On the contrary, 1 hour after BMP-7 injection BMP-7 was detected at high levels in cartilage; the level decreased by 60% 1 day after injection. BMP-7 levels gradually decreased over time, and 7 days after the last injection BMP-7 concentration in treated knees was still higher than that in control knees.

## Discussion

Experimental osteoarthritis induced by ACLT is one of the most widely used models [12-14] and provides temporal progression of cartilage degeneration in the knee. One of the characteristics of this model is that cartilage is affected initially from medial femoral condyle and eventually extends over the lateral femoral condyle [15]. This model alters both the magnitude and distribution of joint forces that are applied to the cartilage surface *in vivo*. Joint instability is the key factor for the onset and/or progression of cartilage degeneration in human osteoarthritis, and this model mimics that mechanism. Because of the instability generated, it is a particularly challenging model for testing structure/disease-modifying osteoarthritis drugs, because the instability that caused the osteoarthritis is still present even as the drug is being applied.

In animal cartilage defect models, several reports have shown that implantation of a scaffold impregnated or mixed with BMP-2 [16] or BMP-7 [17-21] can promote cartilage repair. However, it is expected that a single administration of BMP will not prevent progression of osteoarthritis induced by joint instability. To overcome this potential problem, an osmotic pump was previously used [7]; however, from the standpoint of clinical availability, periodic injections into the knee of BMP-7 would be more attractive. This may also allow the clinician flexibility and options regarding the frequency or duration of the BMP administration, and the potential to adjust the dose delivered into the knee, based on the stage of osteoarthritis.

In this study, the BMP-7 injections were administered at 7-day intervals. Our pharmacokinetic analysis demonstrated that BMP-7 concentration in the cartilage tissue decreased rapidly within 1 day and then the diminution rate decreased, but a measurable level of BMP-7 was maintained for at least 7 days after the injection. Furthermore, immunohistochemical analysis demonstrated higher BMP-7 expression in chondrocytes from the knee 7 days after the injection. There are three possible mechanisms to explain why the effect of BMP-7 on chondrocytes persisted for more than 7 days after knee injection. First, injected BMP-7 remained in the knee joint during activity for over 7 days. Second, exogenous BMP-7 induced endogenous BMP-7 expression in chondrocytes, and then chondrocytes continued to express endogenous BMP-7 in an autocrine/paracrine manner. Third, synovial tissue absorbed injected BMP-7, and then synovial cells expressed endogenous BMP-7 to enhance endogenous BMP-7 expression in chondrocytes [22,23].

In this rabbit ACLT model, we examined the optimal dose of BMP-7 for the prevention of osteoarthritis progression. First, all knees of rabbits that underwent bilateral ACLT were randomly assigned to one of four treatment groups. Although we demonstrated that both 500 ng and 5,000 ng BMP-7 injections prevented the progression of osteoarthritis, there was no significant difference between the two groups. We assume that use of a higher-dose BMP-7 may increase the risk of adverse effects including ectopic cartilage and bone formation, osteophytes, and synovial fibrosis in the knee joint. These doses were much less than those used in other rabbit cartilage studies previously conducted. Sellers and coworkers [16] reported that 5 μg BMP-2 promoted healing of full-thickness defects of articular cartilage. In another study employing a similar design [24], 20 μg BMP-2 was used with a synthetic biodegradable carrier. The amount of 500 ng BMP-7 we used for one injection was only 2.5% to 10% of that in the previous reports.

The results of our first study indicate considerable individual animal variability in osteoarthritis progression after ACLT. We confirmed that the anterior cruciate ligament was completely transected in every knee when the animals were killed, so the injury was uniform in all animals. Therefore, another mechanism must be responsible for the variability. We suspect that differences in activity level between animals and genetic factors may be responsible.

In our second series of experiments, we wished to address the inter-animal variability observed in the first study and to investigate the effect of BMP-7 on osteoarthritis progression in a stricter manner. Therefore, we tested both treatments (500 ng BMP-7 and PBS alone) in the same animal, with each knee receiving a different treatment. This matched pair analyses demonstrated that both macroscopic and microscopic scores were better in the BMP-7 injected knees than in the contralateral control knees in all rabbits at 12 weeks. This provides further evidence that BMP-7 is effective in preventing OA progression and that the previously observed results were probably not due to the individual variability of rabbits.

The initial pathological change in osteoarthritis is characterized by a depletion of the cartilage extracellular matrix. Intra-articular treatment with BMP-7 may enhance the synthesis of new cartilage matrix, possibly preventing further degeneration. Several *in vitro *studies indicated that BMP-7 promotes the production of type II collagen and proteoglycans in normal chondrocytes [1,4]. Even chondrocytes from osteoarthritic patients have been shown to retain their ability to respond to BMP-7, which also upregulates anabolic gene expression in cartilage [2,3,5].

In consideration of drug efficacy and future clinical use, weekly injections are an attractive option; however, further examination is needed to determine the optimal duration of the BMP-7 injection therapy for the osteoarthritic knee, based on several additional *in vivo *studies.

## Conclusion

Weekly intra-articular injections of BMP-7 inhibited osteoarthritis progression. BMP-7 concentration and expression in cartilage were still elevated 7 days after BMP-7 injection. No obvious adverse effects resulted from repeated intra-articular injections of BMP-7.

## Abbreviations

ACTL: anterior cruciate ligament transection; BMP: bone morphogenetic protein; IPF: infrapatellar fat pad; OARSI: Osteoarthritis Research Society International; PBS: phosphate-buffered saline.

## Competing interests

The authors declare that they have no competing interests.

## Authors' contributions

MH carried out the animal experiments, analyzed the results, and drafted the manuscript. YJ and TMo participated in the evaluation of the results. TMu designed the initial plan for the study and participated in the evaluation of the results. IS conducted the experiments, participated in the evaluation, and completed the final manuscript. All authors read and approved the final manuscript.
